# Point-of-Care Testing for Infectious Diseases: Diversity, Complexity, and Barriers in Low- And Middle-Income Countries

**DOI:** 10.1371/journal.pmed.1001306

**Published:** 2012-09-04

**Authors:** Nitika Pant Pai, Caroline Vadnais, Claudia Denkinger, Nora Engel, Madhukar Pai

**Affiliations:** 1 Division of Clinical Epidemiology, Department of Medicine, McGill University, Montreal, Canada; 2 Respiratory Epidemiology & Clinical Research Unit, Montreal Chest Institute, Montreal, Canada; 3 Division of Infectious Diseases, Beth Israel Deaconess Medical Center, Boston, Massachusetts, United States of America; 4 Department of Health, Ethics and Society/Caphri, Faculty of Health, Medicine and Life Sciences, Maastricht University, The Netherlands; 5 Department of Epidemiology & Biostatistics, McGill University, Montreal, Canada

## Abstract

Madhukar Pai and colleagues discuss a framework for envisioning how point-of-care testing can be applied to infectious diseases in low- and middle-income countries.

**Summary Points**

- Enthusiasm and hope are increasing around point-of-care (POC) diagnostics for diseases of global health importance.
- The mere availability of rapid or simple tests does not automatically ensure their adoption or scale-up. A range of barriers prevent the successful use of POC testing—economic, regulatory, and policy-related, as well as user/provider perceptions and cultural barriers.
- Technology as such does not define a POC test. Rather, it is the successful use at the POC that defines a diagnostic process as POC testing. Thus, the focus must be on POC testing programs, rather than POC technologies.
- We discuss a framework that envisions POC testing as a spectrum of technologies (simplest to more sophisticated), users (lay persons to highly trained), and settings (homes, communities, clinics, peripheral laboratories, and hospitals).
- A deeper appreciation of this diversity in target product profiles, and likely barriers in each setting, might help test developers and public health managers to identify the most impactful product and delivery model.

## The Promise of Point-of-Care Testing

Point-of-care (POC) tests have the potential to improve the management of infectious diseases, especially in resource-limited settings where health care infrastructure is weak, and access to quality and timely medical care is a challenge [1],[2]. These tests offer rapid results, allowing for timely initiation of appropriate therapy, and/or facilitation of linkages to care and referral. Most importantly, POC tests can be simple enough to be used at the primary care level and in remote settings with no laboratory infrastructure. POC tests can potentially empower patients to self-test in the privacy of their homes, especially for stigmatized diseases such as HIV [3]. In fact, home-based, over-the-counter HIV testing was approved in July 2012 by the Food and Drug Administration in the United States [4].

Several agencies, notably, the Bill & Melinda Gates Foundation and Grand Challenges Canada, have recently announced grants for the development of new POC diagnostics for global health. Several million dollars are being invested in this area, and substantial enthusiasm and hope are increasing around POC diagnostics. Efforts are also underway to engage diagnostic and biotech companies in emerging economies such as India and China in developing new and affordable diagnostics for TB and HIV [5]. Furthermore, donors such as UNITAID clearly value the importance of good diagnostics and are actively supporting projects on TB, HIV, and malaria diagnostics [6].

In this context, various stakeholders need deeper insights into the challenges for use and scale-up of POC testing, and a framework for thinking about the diversity of product profiles involved in POC testing, including where and how POC testing can be implemented successfully, what barriers need to be overcome, and what characteristics are necessary for impact.

## Diversity of Definitions and Target Product Profiles within POC Testing

According to one textbook, point-of-care testing (POCT) can be defined as the “provision of a test when the result will be used to make a decision and to take appropriate action, which will lead to an improved health outcome” [7]. Another definition is: “patient specimens assayed at or near the patient with the assumption that test results will be available instantly or in a very short timeframe to assist caregivers with immediate diagnosis and/or clinical intervention” [8]. However, there are dozens of definitions of POCT and no accepted universal definition [9]. Regardless of the exact definition, we believe that the most critical elements of POCT are rapid turn-around and communication of results to guide clinical decisions and completion of testing and follow-up action in the same clinical encounter [10]–[12].

Rapid turn-around of results is critical for the test results to impact clinical management (e.g., triage, referral, treatment decisions, decision to discharge, etc.). Indeed, without a clear link to a treatment or counseling plan, test results, even if rapid, are unlikely to have an impact [13]. “Rapid” can range from within seconds, to minutes, to a few hours (“while the patient waits”). At the least, results “on the same day” can still help disposition of clients with a clear plan (e.g., initiation of anti-tuberculosis or anti-retroviral therapy). Convenience to patients and care providers mainly derives from the fact that the POC diagnostic process is completed “in the same clinical encounter,” as compared to conventional testing where clients/patients may not come back for testing or go far away (or wait long) for testing. One of the biggest concerns about conventional laboratory-based testing is the long turn-around times and delays, and the resultant loss of patients from the testing and treatment pathway. This is, for example, a well-recognized concern with conventional sputum smear microscopy for tuberculosis (TB) [14], and laboratory-based CD4 and viral load testing for HIV [15].

We suggest that the technology as such does not define a POC test nor determine its use at the POC. Rather, it is the successful use at the POC that defines a diagnostic process as POC testing. So, it may be best to think of POC testing programs, rather than POC tests. It is how the tests are deployed or implemented in a health system that defines a POC testing program. For example, one could implement a rapid diagnostic test (RDT) or dipstick in a reference laboratory, and that will not be a POCT program. Indeed, laboratories in resource-limited countries often use RDTs, but results are often delivered after days. On the other hand, one could implement a molecular test in an out-patient clinic and successfully allow POC usage. The Xpert MTB/RIF test based on GeneXpert technology (Cepheid Inc) is one such technology that can potentially be implemented in TB clinic settings and peripheral laboratories [12].

Thus, systems for rapid reporting of test results to care providers, and a mechanism to link test results to appropriate counseling and treatment are as important as the technology itself. If systems for reporting the results and follow-up care are not in place, then POC testing is unlikely to have an impact on clinical or public health outcomes [13]. Also, POCT programs require viable business models to ensure that they are sustainable in the real world and will actually get used. This means POC testing must fit within real-world workflow patterns and economic/incentive structures.

It is widely believed that POC tests should be equipment free, simple RDTs (that is, those that meet the “ASSURED” criteria: affordable, sensitive, specific, user friendly, rapid and robust, equipment-free, and delivered [2]) and always be done outside of laboratories and hospitals by non-laboratorians. Such criteria were probably necessary when RDTs were introduced, but in today's context, they impose artificial restrictions on the concept of POCT. POC testing done at the point of clinical contact is preferable but not required, so long as a system is in place for rapid reporting of results that can inform clinical decisions. For example, testing at a peripheral laboratory attached to (or near) a clinic or hospital can still allow for POCT. Such POC testing can be done by hospital staff in emergency rooms, operating rooms, intensive care units, and labour wards, without waiting for results to come from laboratories, and this means POCT can also occur in hospital settings [16]. Indeed, there are successful examples of POC testing by non-laboratorians in hospital-based settings such as emergency rooms (e.g., rapid influenza testing [17]) and labour wards (e.g., rapid HIV testing to reduce mother-to-child transmission [18]).

Also, restricting POCT to really cheap and equipment-free tests (e.g., RDTs—also called “first-generation POC tests”) imposes barriers for use of newer technologies such as cartridge-based POC nucleic acid amplification tests (NAATs; “second-generation POC tests”) and hand-held devices such as mobile phone-based technologies (“third-generation POCTs”) [19]. These newer POCTs may not be as cheap and equipment free as RDTs and dipsticks, but may prove to be very impactful and cost-effective in the right context [20].

**Figure 1:**
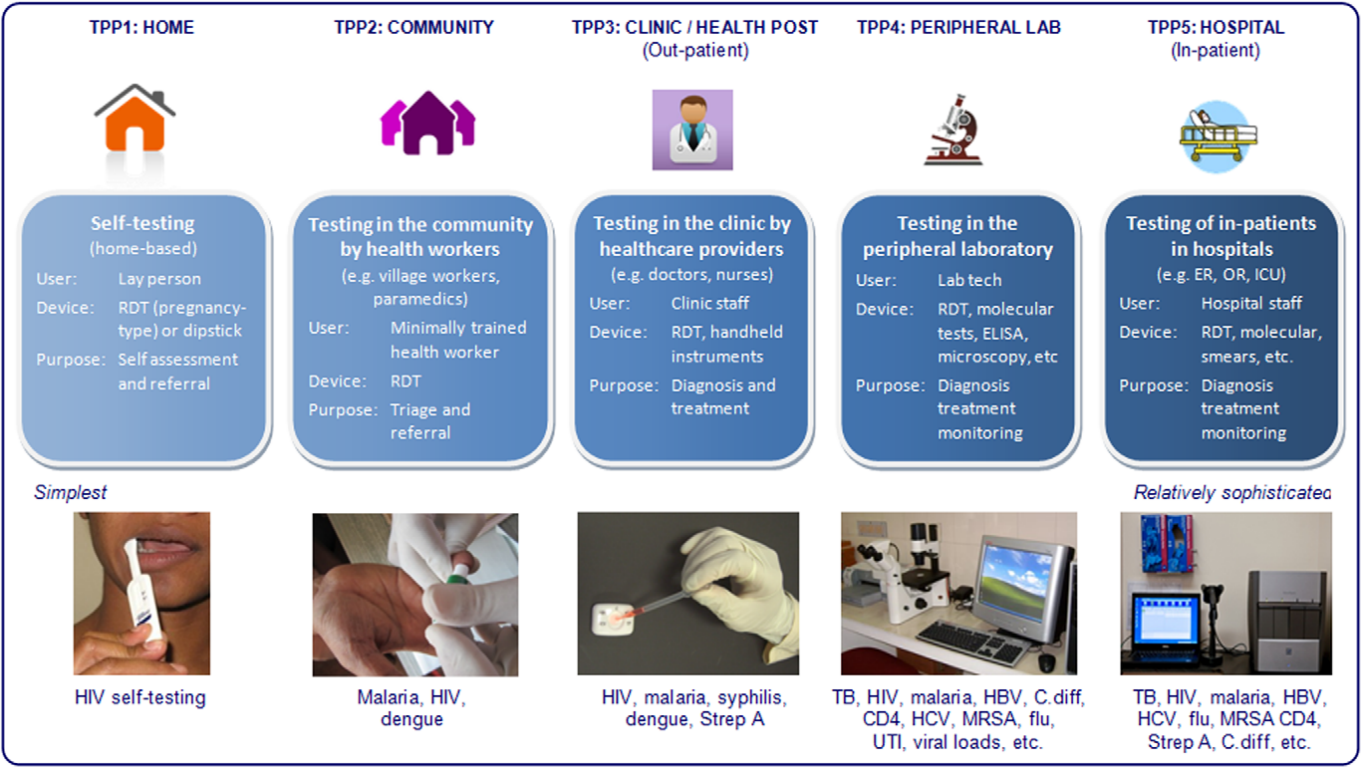
Diversity of target product profiles, users, and settings within the spectrum of POC testing. HBV, hepatitis B virus; HCV, hepatitis C virus; UTI, urinary tract infection; MRSA, methicillin-resistant staphylococcus aureus; C. diff, clostridium difficile; RDT, rapid diagnostic test; Strep A, group A streptococcus.

Thus, we propose that it is easier to think of POC testing as a spectrum of technologies (simplest to more sophisticated), users (lay persons to highly trained), and settings (homes to hospitals). This diversity of target product profiles (TPPs) within POCT is illustrated in Figure 1. This framework shows that POCT can be done in at least five distinct settings: homes (TPP1), communities (TPP2), clinics (TPP3), peripheral laboratories (TPP4), and hospitals (TPP5). Unique barriers may operate at each level, and prevent the adoption and use of POCTs. As shown in the schematic, there are several examples of POC tests in each of these settings. The relative importance of these settings may vary by country, and also within a country, where there may be important differences in public versus private sectors, and rural versus urban areas.

In the framework that we propose (Figure 1), the type of device does not define a POC test. As mentioned, POC tests can range from the simplest dipsticks to sophisticated automated molecular tests, portable analysers, and imaging systems. The same lateral flow assay, for example, could be used across all TPPs. Hence, the device does not automatically define the TPP, although some types of devices will immediately rule out some TPPs or users. For example, conventional ELISA cannot be performed by lower level health workers or even doctors. Microscopy is another technology that requires a trained user and quality assurance mechanism—this restricts the technology to laboratories and hospitals. In general, even the simplest molecular tests require basic infrastructure such as power supply and temperature control, and are therefore unlikely to be used in TPP1–TPP3 in resource-limited countries.

Also, the end-user of the test does not automatically define a POC test. The same device (e.g., lateral flow assay), can be performed by several users across the TPPs—from untrained (lay) people, to community health workers, to nurses, to doctors, and laboratory technicians. Rapid oral-fluid–based HIV tests are a good example of such a test [9], because it now spans the entire spectrum—from in-home testing to hospital-based testing. So, the actual user does not immediately identify the TPP, although targeting the end-user helps narrow down the type of TPP needed (e.g., lay person or lower level health worker necessarily means the simplest type of device and the most robust design). For example, in-home testing for HIV demands the simplest type of device, along the lines of a pregnancy test, and may also require telephonic counseling and support services [4],[21],[22].

Depending on the end-user and the actual setting, the purpose of POC testing may vary—from triage and referral, to diagnosis, treatment, and monitoring. Some POC test users may not be empowered to prescribe medicines, while others can use POCT results for treatment. This has implications for test developers. A test that is intended for triage and referral can have different accuracy (e.g., lower specificity), compared to a test that is used to make treatment decisions [23].

## The Need to Understand Barriers for POC Testing

The best POCT will not have any impact unless it is widely used and followed-up with appropriate treatment interventions [24]. The mere availability of rapid or simple tests does not automatically ensure their adoption or scale-up [24]. This observation is evident from the global experience with rapid HIV tests and malaria RDTs [5],[25]–[28]. In India, for example, simple RDTs are available for a variety of diseases (e.g., HIV, malaria, dengue, syphilis, hepatitis), are quite inexpensive (US$1 per test or less), and some meet all the ASSURED criteria. However, these RDTs are often not used in community or clinic settings to make clinical decisions (with the possible exception of pregnancy tests and possibly HIV and malaria RDTs). It appears that very little POCT occurs in homes, communities, or clinics (TPP1–TPP3). Testing predominantly takes place in laboratories and hospitals (TPP4 and TPP5). In fact, small, stand-alone laboratories are the biggest consumers of RDTs.

What are the most important barriers to widespread use of POCT at lower levels of the health care delivery system, where we hope POC testing will reduce diagnostic delays and interrupt transmission? On the basis of our observations and the published literature [2],[11],[26]–[30], we believe there are a variety of barriers to successful use of POCT—from economic, regulatory, and policy-related barriers to user/provider perceptions and cultural barriers. Table 1 provides illustrative examples of these barriers. Some barriers are generic, while others are restricted to a specific TPP or setting. Barriers for POC testing may depend on the country, and may also differ across public versus private, and urban versus rural settings. In addition, some barriers may be disease-specific, while others will apply to all types of tests. For example, stigma and confidentiality may be important barriers for HIV testing [26],[28],[31], while they may be less relevant with malaria or dengue testing.

**Table 1:** Barriers to adoption and scale-up of POC technologies.

Barrier for POCT	Example
**Economic**	It may be more expensive to place test instruments at the POC, as compared to laboratories. Some POCTs may be priced at a level that is unaffordable in many countries. Private care providers may receive incentives from laboratories for each test that they order; this means they can earn more by sending their patients to labs rather than do any POC testing.
**Policy-related**	Existing guidelines and policy documents may not provide clear recommendations on how to include POC tests in algorithms that are in place. Lack of a strong evidence-base on POCTs can result in weak evidence and uncertain policy recommendations.
**Regulatory**	Poor regulation of diagnostics may result in easy availability of suboptimal and poor quality rapid tests on the market; this makes it challenging to scale up validated POCTs.
**Laboratory capacity**	Some POCTs may require peripheral labs with sufficient capacity to run them (e.g., nucleic acid amplification tests). Poor laboratory capacity poses a barrier for scale-up of such technologies.
**Infrastructure**	Clinics and primary care centers often lack infrastructure such as constant power supply, refrigerators, storage space, waste disposal units, phlebotomy supplies, and temperature control; this makes it hard to implement some types of POCTs.
**Quality control and quality assurance**	Even simple POC tests require quality assurance and training before they can be performed. Primary care providers may not have the expertise or training to do them with quality assurance.
**Work-flow balance**	Staff shortages and high workload may reduce uptake of POCT. Health care providers are overburdened with a high volume of patients, and work-flow and time constraints do not permit easy use of POC tests.
**Training**	Unqualified and informal care providers may lack the knowledge and training needed to implement even simple RDTs. Erroneous results then erode the health system's faith in POCT. Lack of continuous, ongoing proficiency training can result in diminishing performance of POCT programs.
**Supply chain**	Supply chain deficiencies can lead to suboptimal or poor quality POC tests, which, in turn, may discredit POCT.
**Infection risk**	Health providers may be unwilling to do tests that may expose health care workers to the risk of infection.
**Administrative/operational**	It is not easy for health providers to seek reimbursement from insurance providers and third-party payers when POC tests are used in community or home settings.
**Technical/medical**	Doctors and front-line care providers in some settings may prefer clinical diagnosis and empiric treatment over diagnostic certainty. Widespread empiric treatment of common diseases reduces the felt need for any testing, POCT or otherwise.
**Awareness**	Health workers and care providers may not be aware of the various tests that are now available for POC use. Thus, they may still refer their patients to laboratories for testing.
**Health system-related**	Laboratory professionals in hospitals and larger health care facilities are opposed about any testing that is done outside of lab settings. They fear this will impact their own business, and they also worry about relinquishing control over testing.
**Fit with user needs**	Available rapid tests are often single disease focused when primary care providers are more worried about syndromes of unknown etiology (e.g., febrile illness, chronic cough). So, available tests may not quite meet user needs.
**Cultural/societal**	Perceived lack of confidentiality and stigma may reduce acceptance of POC testing in the community (e.g., HIV rapid tests).

## Why Do We Need to Understand the Diagnostic Ecosystem in Countries?

It is particularly important to look beyond the technology, and understand current diagnostic practices and the health systems within which POC testing has to get scaled up. At the country level, POC diagnostics ultimately need to be integrated within health systems, supported by financing (who will pay and how much?), incentives (do various stakeholders benefit from the economics?), training and information and communications technology (ICT). These other factors (“the business model”) may be as important as the POCT itself and need to be taken into account when developing tests [32].

Indeed, the best POCT without a good business model is unlikely to get scaled up, while, paradoxically, inaccurate tests can become popular because of economic reasons, as illustrated by the apparent market success of inaccurate TB serological tests in many developing countries [33],[34]. In India, we have shown that although serological TB tests are inaccurate, various players along the value chain profit from their use, and this sustains a market for these tests [34].

ICT, when combined with POC, can help expand care to lower tiers of the health care delivery system, all the way to home-based, self-testing [30]. Thus, the rapid expansion of mobile telephony makes telephonic counseling and rapid reporting of results (to patients as well as to public health programs) feasible in many settings. Diagnostic devices linked with mobile phones can also allow for automatic data capture, external quality assurance, and proficiency testing. Smart phones can also provide decision support to health workers on what follow-up action is necessary after testing. Indeed, new TPPs and business models are now feasible, thanks to the rapid expansion of ICT. Interestingly, the mobile phone itself is becoming a POC testing device, and this is a vibrant area for incentive prizes [35].

In India, the diagnostic ecosystem is worrisome with systematic market failures throughout the value chain for diagnostics—private doctors receiving payments or incentives for tests ordered, over-reliance on useless tests, and under-use of good diagnostics [33],[34],[36]–[38]. There is little quality assurance for laboratories in India and private labs offer tests of doubtful value. Laboratories in the public health sector suffer from poor infrastructure and limited capacity, while dealing with massive volumes. The regulatory framework for in vitro diagnostics in India is weak and most diagnostics do not undergo rigorous validation before approval [34]. As a result a large number of inaccurate and ineffective products can be found on the market; many of these are imported, but not approved by the US Food and Drug Administration (FDA) or other such credible regulatory bodies outside of India [34]. In fact, the Government of India has recently banned TB serological antibody tests, which are widely used in the private sector [34].

It is within this context that we need to understand the barriers for use of POC diagnostics in India. Based on our work in India on TB and HIV diagnostics [3],[18],[29],[33],[34],[36],[37],[39]–[43], we have identified several potential barriers to implementation of POC tests by primary care providers (Table 2). Additional barriers may operate at the community level in India. Much of the community-level health care in India is done by village health nurses, auxiliary nurse midwives, and Accredited Social Health Activist workers, under the National Rural Health Mission. These workers are generally not well trained or empowered to adequately use POC tests or prescribe drugs on the basis of test results (with some exceptions). In fact, the medical lobby in India vigorously prevents any move to empower health workers to prescribe drugs. Furthermore, these community health workers are heavily burdened with paperwork associated with maternal and child health-related programs and often do not have the time to conduct any testing. Furthermore, the incidence of many diseases is quite low at the community level, and this might reduce the motivation and resources for community-based testing.

**Table 2:** Barriers for use of point-of-care tests in India.

Category	Potential Reasons Why POC Tests Are Not Being Used at the POC
**Technical, administrative, and operational**	**Widespread empiricism in clinical practice:** Doctors and front-line care providers in India generally prefer clinical diagnosis and empiric treatment (e.g., with broad spectrum antibiotics) over diagnostic certainty.
	**Work-flow, time constraints, and patient volume:** Care providers are overburdened with a high volume of patients, and because average consultation times last for just a few minutes, the work-flow and time constraints do not permit POC testing. In the time it takes to do the POC test and read it, providers could see several waiting patients and that is more important for their popularity and reputation.
	**Expertise and self-confidence:** Care providers in India have a diversity of backgrounds—ranging from MBBS-trained medical doctors, to unqualified providers, and those with training in alternative medicine. Thus, the ability of these providers to do any clinical testing is highly variable. Unqualified practitioners and those with non-medical backgrounds may lack the skills (or the confidence) to perform and interpret tests (even if they are simple to use). Even MBBS doctors may not want to take on the responsibility of doing and interpreting test results.
	**Infrastructure and support staff:** A large number of care providers in India practice medicine from small, single-room clinics, with very little space for even a small side laboratory. They often practice alone with no support staff (e.g., nurse). This makes it difficult to implement even simple lateral flow tests. Clinics often lack basic equipment such as refrigerators, storage space, waste disposal units, phlebotomy supplies, temperature logs, etc.
	**Quality assurance training:** Care providers may not have the expertise nor training to do testing with quality assurance (e.g., run positive/negative controls).
	**Fit with user needs:** Care providers manage several commonly encountered infections (e.g., TB, malaria, dengue, and influenza), and it is not feasible for them to remember all the standard operating procedures of these tests, nor is it possible for them to perform several rapid tests on the same patients to work through their differential diagnoses.
	**Tests for multiple infections:** Available rapid tests are often single disease focused (e.g., malaria RDTs) when primary care providers are more worried about syndromes of unknown etiology (e.g., febrile illness, chronic cough, diarrhea). In the absence of multiplexed POC tests for a panel of related infections, they find it hard to do multiple POC tests at the POC.
	**Investments at the primary care provider level:** At the level of the single primary care provider, there is insufficient volume of each disease to make it worth their while to stock multiple RDTs. Also, care providers do not like to make any capital investments, especially in small centers.
	**Reputation of POCTs:** Easy availability of suboptimal and poor quality rapid tests in the market may have resulted in lack of faith in them—care providers consider rapid tests to be inferior to conventional tests.
**Health system-related**	**Awareness of POCTs:** Doctors and care providers may not be aware of the various POC tests that are now available for POC use.
	**Professional exclusivity:** Laboratory professionals in hospitals and larger health care facilities are opposed to any testing that is done outside of lab settings. They fear this will impact their own business, and they also worry about relinquishing control over testing. Laboratory professions do not believe that doctors or health care workers can do POC tests with quality assurance.
	**Monitoring and tracking outside of labs:** Health care delivery systems struggle to audit and monitor the use of POC tests in settings outside of the laboratory. In India, medical records are poorly managed in most settings.
	**Impact on hospital attached labs:** Hospitals and medical establishments often have their own laboratories, and to generate business for their labs, they prefer that doctors refer patients to these laboratories, instead of doing testing by themselves.
	**Investment in post-test counselling:** Health care systems may be unwilling to invest in counselors who can provide post-test counseling services in clinic settings.
	**Weak capacity to absorb POC tests and insufficient treatment availability:** Implementation of POCT might increase workload and demand for treatment services. Limited resources (e.g., drugs) and human resources might diminish enthusiasm for POCT.
**Economic**	**Clinical relationship and exchange of money:** The usual practice in India is for patients to pay someone else (e.g., a secretary or nurse) rather than the doctor directly. If the doctor were to perform and read the POC test, it will be awkward for them to take money from patients (unless support staff are involved).
	**Referral fees and incentives:** Private care providers often receive referral fees (i.e., incentives) from laboratories for each test that they order; this in turn greatly limits their financial incentive to directly conduct tests.
	**Patients**' **willingness to pay:** While patients may be willing to pay for tests done at a laboratory, they may be unwilling to directly pay the doctor for testing costs, beyond the consultation fees that they are already paying.
	**Affordability of POCTs:** Poor patients may not be able to afford any testing. They may instead prefer a prescription for drugs (which may be less expensive and more easily available compared to tests).
	**Traceability for insurance reimbursements:** It is not easy for health providers to seek reimbursement from insurance providers and third-party payers when POC tests are used in office, community or home settings, with no invoice getting generated for each test.
	**Bypass of medical consultation and diagnostic process:** Patients seeking quick relief from symptoms may directly buy over-the-counter antibiotics from pharmacies, and thereby completely bypass the medical consultation process.
	**Mark-up on POCTs:** Laboratories that offer POC tests may demand a high price for these tests, making them unaffordable for poor patients.

In South Africa, discussions with TB/HIV experts suggest that much of the POC testing currently occurs in laboratories and hospitals (TPP4 and TPP5). For example, the scale-up of Xpert MTB/RIF in South Africa is happening via the National Health Laboratory Service (NHLS) network of laboratories. At the clinic level (TPP3), tests for infections like HIV, syphilis, and malaria are widely used. At the community level (TPP2), HIV rapid tests are the most widely used POC tests. HIV self-testing is known to happen at home in South Africa (TPP1), especially among health care workers who avoid conventional voluntary counseling and testing (VCT) to protect their confidentiality.

South African experts identified several potential barriers for POCT in their setting. For example, laboratory professions are concerned about widespread use of POC tests for many reasons: (1) NHLS cannot control or provide oversight to any testing that is done outside of the NHLS lab network; (2) it is not clear which agency will accept ownership of a POC testing program in South Africa—who will conduct training, quality assurance, maintenance; (3) it is unclear who will provide overall management of a decentralized POC testing program at the level of communities and clinics. Cost can also be a major barrier for POCT—a recent study from South Africa suggests that placing the Xpert MTB/RIF test at the POC will be substantially more expensive than placing the instruments in the NHLS laboratories [44].

## Overcoming Barriers to POCT Programs

POC testing holds a lot of promise, but it is important to understand the complexity and diversity of POCT, and identify the biggest barriers to successful implementation of POCT programs. This step is critical for development and scale-up of POCTs, because it will allow test developers and public health programs to target the TPP that is most likely to succeed and has the most impact.

The framework we have proposed may have utility in shaping many of the ongoing efforts to develop and deploy POC tests for global health. Firstly, test developers and manufacturers need to understand the real-world context (e.g., conditions, settings, users, resources) within which tests need to get scaled up. Only then can TPPs by test developers match the TPPs required by public health programs. Indeed, technologies may need to be designed in resource-limited settings (“frugal or reverse innovation”), from the ground up, to ensure that they are robust, field-tested in a variety of conditions, have built-in capacity for reporting/notification, and appropriately priced. Secondly, donors and funding agencies must consider the downstream implications of the health technologies that they are funding, and ensure that product development initiatives are simultaneously coordinated with pricing and delivery mechanisms, supported by innovative business models for scale-up. Lastly, health care managers must invest in POCT programs, rather than merely purchase rapid tests, and ensure the mechanisms are put in place for quality assurance, reporting of results, notification of cases, and initiation of action on the results of the tests. Only then will the true benefits of POC testing be realized.
